# Optimization of an experimental study of cationic Pb metal adsorption by resin polymer

**DOI:** 10.1038/s41598-023-46967-3

**Published:** 2023-11-16

**Authors:** Jaouad Bensalah, Ghizlane Doumane, Oumayma Iraqi, Ahmed A. Elhenawy, Hanae Ouaddari, Mohammad K. Okla, Hiba-Allah Nafidi, Youssouf Ali younous, Mohammed Bourhia, Amar Habsaoui

**Affiliations:** 1 Laboratory of Materials Advanced and Engineering Process, Department of Chemistry, Faculty of Sciences, University Ibn Tofaïl, B.P. 133, 14000 Kenitra, Morocco; 2 Faculty of Science, Al-Azhar University, Cairo, Egypt; 3 Chemistry Platform, UATRS, National Center for Scientific and Technical Research (CNRST), Rabat, Morocco; 4 Botany and Microbiology Department, College of Science, King Saud University, P.O. Box 2455, 11451 Riyadh, Saudi Arabia; 5 Department of Food Science, Faculty of Agricultural and Food Sciences, Laval University, 2325, Quebec City, QC G1V 0A6 Canada; 6 Evangelical College, BP 1200, N’Djamena, Chad; 7 Department of Chemistry and Biochemistry, Faculty of Medicine and Pharmacy, Ibn Zohr University, 70000 Laayoune, Morocco

**Keywords:** Chemistry, Materials science

## Abstract

To eliminate lead (Pb) ions from metallic solutions, the cationic resin in solid form was utilized. The characterization of the adsorbent was performed using GTA/GTD, SEM spectroscopy, and EDX analysis. The results of these analyses provided insights into the structure and composition of the resin. The removal of Pb (II) ions was found to be highly dependent on various parameters. Firstly, the pH of the metal solution played a crucial role, as the adsorption capacity increased with the pH of the solution, at a maximum equal to (R = 84.78%), at a pH = 8.0. Additionally, the concentration of Pb (II) ions present in the solution influenced the adsorption technique’s capacity, with higher concentrations leading to increased adsorption, analysis overhead of high concentration present (100 mg L^−1^) of the metal lead (II) study, a saturation corresponding a plateau to the resin polymeric saturation is 93.18 mg g^−1^. To determine the optimal mass of the resin adsorbent, a study was conducted to maximize the removal of Pb (II) ions, at the mass 1.0 g showed that the proportion of inorganic pollutants removed from Pb (II) is entirely qualitative (100%). Furthermore, the effect of temperature on the adsorption process was investigated. It was observed that the rate of the Pb (II) adsorption process decreased as the temperature increased. Kinetic studies were performed to gain further insights into the adsorption process. Pseudo-first-order and pseudo-second-order models, along with the intra-particle diffusion model, were utilized for this purpose. The results indicated that the adsorption process was fast, as evidenced by the findings from the pseudo-second-order study. The saturation technical process was studied, employing several different isothermal models, including Langmuir, Freundlich, and Temkin. Among these models, the Langmuir model was found to best describe the phenomenon of lead metal adsorption by the resin polymeric, is equal to 11.23 mg g^−1^, with the experimental value precisely (R^2^ = 0.999). Finally, various thermodynamic techniques were applied to analyze the adsorption process. The thermodynamic parameters such as ΔG° (− 9.78 to − 9.27 kJ mol^−1^), ΔH° (14.85 kJ mol^−1^), and ΔS° (0.017 kJ mol^−1^) were determined. These values indicated that the adsorption process was endothermic and spontaneous, further emphasizing its impetuous nature. The results of the molecular dynamics calculations demonstrated that amino groups are very important in defining the characteristics of cation adsorption. We conclude that this new adsorbent has the potential to significantly improve the process of regularly removing heavy metal ions from wastewater.

## Introduction

The presence of heavy metals in aquatic environments is a major concern for scientists and human health due to their release and toxic nature. These metals are often resistant to degradation and difficult to detoxify, making their presence in recipient waters problematic. The effective treatment and disposal of wastewater containing heavy metals remain a challenge for both industries and the aquatic environment, as cost-effective alternatives are yet to be found^1–3^.

One particular heavy metal of concern is lead (Pb), which has been found in natural water sources due to various activities such as battery storage, mining, lead smelting, investments, and ceramic glazing industries. The three-dimensional polymer network structure which is composed of hydrophilic polymer molecules endows it with strong hydrophilicity, making it absorb a large amount of water in an aqueous solution, Therefore, solid polymeric has been used as adsorbent for heavy metal ions widely in the past few years. To address this issue, the adsorption process is commonly used, where adsorbents are utilized to remove micropollutants from water^4,5^. This technique is preferred due to its simplicity, effectiveness, viability, and social acceptance. In this study, a cationic resin is employed as the adsorbent for lead metal in aqueous medium. The kinetics of lead (Pb) elimination using this cationic resin are investigated, and it is important to accurately describe the adsorption kinetics. Various models are used to compare the expected adsorption techniques and the experimental behavior of the resin in different conditions^6–8^. The utilization of adsorption models allows for the optimization of adsorption techniques, understanding the relationship between the surface morphology of the cationic resin and the adsorption outcomes, determining adsorption capacities, and efficiently designing adsorption systems. These models help in identifying the most effective parameters and conditions for the adsorption process, leading to better treatment outcomes^2,4–7^.

## Procedure

### Reagents

They have solutions in which Pb (NO_3_)_2_(H_2_O)_5_ (99.9%) is used as a source of Pb (II) ions. This compound is employed to effectively bind metal ions in metallic solutions. All these solutions are prepared in the laboratory with distilled water.

### Adsorbent resin polymeric

The resin polymeric beads are supplied by the National Center for Scientific and Technical Research (CNRST) in Rabat-Morocco. The material was previously used in the process of water demineralization.

In order to remove metallic traces, the resin polymeric was rinsed heated at 60 °C for 3 h. The product was rinsed with distilled water and dried. The resin was demineralized with 1.0 M HCl (97%) hydrochloric acid for 24 h at a ratio of 100 ml: 1 g (w/v) at room temperature and kept for 24 h. Subsequently, after several successive washes, a clean filtrate with a pH of approximately 5 was obtained. The material that was studied was dried in the oven at T = 60 °C for 24 h. Finally, the resin polymeric is stored in distilled water until it is ready for use in the adsorption experiments.

The low exchange resin polymeric was used in this study to characterize the phenomenon of Pb(II) ions as the adsorbent material, which is a type of an adsorbent polymeric with the chemical formula C_23_H_37_Cl_2_N_3_O and the chemical designation: (4-benzyl-methyl-1,4-diazepan-1-yl)-(9-amino-3-bicyclo [1, 3, 3] nonane)-(4-benzyl-methyl-1,4-diazepan-1-yl)methane, the molar mass 442.5 g mol^−1^. In this study, we have employed the same methodology and techniques that are commonly used by researchers in the field^9,10^. Aqueous of the ICP standards calibration in 5% of the HNO_3_ acid, were prepared on the lead ion Pb (II) ions with the chemical formula Pb(NO_3_)_2_(H_2_O)_5_ of analysis from Carlo Erba multi elements standards and the chemical designation: Unhydrated lead nitrate with the molar mass 331.2 g mol^−1^, and the Q_C_ samples were prepared from Sigma Aldrich mono element Standard. The measurement was carried out at λ = 220.353 nm, which corresponds to the highest absorbance of the lead ion Pb (II).

### Electrostatic potential and maps/FMO analysis

Conceptual density functional theories (DFT) can be used to support the understanding of the adsorption mechanism for the resin polymeric. T-DFT. B3YlP/6311G quantum chemical calculation was used to analyze FMO.

### Kinetic, isotherm and thermodynamically adsorption and prevalence models

The kinetic study carried out by shaking an optimal mass of the resin polymeric, previously determined, in the aqueous solution of the Pb metal at 20 mg L^−1^. The elimination of the dye followed during 5 h of contact time with the adsorbent. The kinetic models of 1st pseudo order, the pseudo 2sec order, Elovich’s, Intraparticle diffusionand Bangham model’s were utilized to estimate the kinetics of adsorption. On the other hand, the technical adsorption temperature assorted from 298 to 328 K to get the measure of the effect of this various parameters on technical phenomenon and to conclude the various thermodynamic parameters, in the isotherme party is estimed the Langmuir, Freundlich and Temkin moldel’s.

## Experiments adsorption party

### Kinetic of the contact adsorption

In the working mode, 100 L of lead metal (II) ion solution was brought into contact with 0.1 g of the adsorbent organic the resin polymeric at an adjusted pH of 5.5. In a beaker, 0.1 g of the organic resin polymeric is inserted with stirring at a T = 298 K. [Pb] = 20 mg L^−1^. 1 mL of the solution in a phial of 10 mL vials distilled contained water at regular intervals, and initial concentration of this metal study lead remaining in the solution diluted was automatically measured, with the mass molar of the Pb is 207.2 g mol^−1^. The stirring is paused before each withdrawal, and the total solitary volume does not exceed 20% of the V(ml) beginning of this metal lead (II) solution. An AA240FS variable atomic spectrometer absorption used to lead test concentrations (AAS). The quantity of the metal Pb (II) adsorbed at contact time t, The variance between the initial concentration of Pb (Ci) and the instantaneous concentration of Pb (Ce) was used to conduct the calculated capacity (qe) study, Ct was exploited is following in the next Eqs. (1) and (2).

**Table:** 

Equation	Nomenclature
$$\begin{gathered} Q_{e} = \left( {C_{0} - C_{e} } \right) \times \frac{V}{m}{ (1)} \hfill \\ R(\% ) = \frac{{\left( {C_{0} - C_{e} } \right)}}{{C_{0} }} \times 100{ (2)} \hfill \\ \end{gathered}$$	C_0_: Concentrations initial (mg L^−1^)
	C_e_: Concentrations equilibrium study (mg L^−1^)
	m : Mass adsorbed of resin polymeric (g)
	V : Volume study of Pb(II) (mL)

### Kinetic and diffusion models

The experimental data obtained in this study were incorporated into well-established studies and extended models to gain a thorough understanding of Pb (II) solution on adsorbent cationic resin polymeric. Table 1 shows the models used in this investigation.

**Table 1:** Kinetic adsorption of different models exploiting.

Models kinetic	The equations	Parameters	References
Pseudo 1st order	$\frac{{dq_{t} }}{dS}{\text{ = KF}}\left( {q_{e} - q_{t} } \right){ (3)}$ $\log \left( {q_{e} - q_{t} } \right) = \log \left( {q_{e} } \right) - \left( {\frac{{k_{{}} }}{2.203}} \right).t{ (4)}$	q_t_ : Biosorption capability over time (mg g^−1^)	^9^
		q_e_: Biosorption capability of equilibrium study (mg g^−1^)	
		k: Constant study of pseudo 1st order model (g mg^−1^ min^−1^)	
		t: The time expressed in (min)	
Pseudo 2sec order	$\frac{t}{{q_{t} }} = \frac{1}{{kq_{e}^{2} }} + \frac{1}{{q_{e} }} \times {\text{t (5)}}$	k: Constant of 2sec order model present (g mg^−1^ min^−1^)	^10^
Elovich’s Kinetic	$q_{t} = \frac{1}{\beta } \times \ln \left( {\alpha \beta } \right) + \frac{1}{\beta }\ln \left( t \right){ (6) }$	α: The initial rate of the adsorption expressed (mg g^−1^ min^−1^) β: The constant of desorption in grams per milligram	
Intraparticle diffusion model	$q_{t} = K_{i} \times t^{1/2} + \chi_{i} (7)$	q_t_ : The amount of metal adsorbed on the solid phase is expressed in mg g^−1^;	^11^
		Ki: The slope and concentration value of the junction of the graph curves qt against t^0.5^ were used to calculate the intraparticle diffusion coefficient (mg g^−1^ min^-0.5^)	
		C: Diffusion constant (mg g^−1^)	
Bangham model	$\log \log \left( {\frac{{C_{0} }}{{C_{0} - q_{t} \times m}}} \right) = \log \left( {\frac{{K_{B} }}{2.303 \times V}} \right) + \alpha log\;\;(8)$	qt: The quantity of metal adsorbed at time t (mg g^−1^)m : Mass study of adsorbent (g)	^12–14^
		ɑ are K_B_ the constants	

### Thermodynamics adsorption party

The thermodynamic behavior of heavy metal in this highly technical process in specific solutions is crucial for properly comprehending this type of mechanism molecular involved in the adsorption process. The thermodynamics of various parameters, including energetics such as ΔG° (Gibbs free energy), ΔH° (enthalpy), and ΔS° (entropy), were investigated. The various values of ΔH° and ΔS° correspond to the coefficient and intercept obtained from the linear regression analysis of the graph plotting Ln (K_L_) versus 1/T. This graph is commonly used to study the relationship between the natural logarithm of the equilibrium constant (K_L_) and the reciprocal of temperature (1/T). Table 2 shows the equations that were used to compute the ΔG° values.

**Table 2:** In this work, we have used the thermodynamic equations below.

Nam of the parameters study	Equations	Parameters	Reference
Energy of Gibbs	$$\begin{gathered} {\text{ Ln (K}}_{L} {) = }\frac{\Delta S}{R} - \frac{\Delta H}{{RT}} \, \left( 9 \right) \hfill \\ \Delta G = \Delta H - T\Delta S \, \left( {10} \right) \hfill \\ \end{gathered}$$	∆G°/∆H°: The energy study of Gibbs free and Enthalpy successively (KJ mol^-1^)	^15^

### Isotherm study of adsorption

In the isotherm’s adsorption party, the concentrations of inorganic solutions in metal Pb, which varied range [Pb] = 20 mg L^−1^ at [Pb] = 200 mg L^−1^, the adsorption time repaired to that established in this test kinetic. After ranging the determined time contact, the various samples exploited by technical analyzed ICP.

The relationship between Pb (II) introduces in the solution, because of the research of this isothermal party technique. They adsorbed on the resin adsorbent polymeric when these two equilibria are investigated. This research enhanced our comprehension of specific characteristics that could help the adsorption design processes as well as the nature and mechanism molecular involved in various technics approaches. Langmuir, Temkin, and Freundlich were the isothermal models studied can see in Table 3.

**Table 3:** Adsorption isotherm models exploited.

Isotherm Model	Equations Linearized	Parameters	Reference
Langmuir	$\frac{{C_{e} }}{{Q_{e} }}{ = }\frac{{C_{e} }}{{Q_{m} }}{ + }\frac{1}{{\left( {K_{L} .Q_{m} } \right)}} \,$ (11)	Qe: Capacity adsorbed of adsorbent (mg g^−1^)	^16^
		Ce: The concentration study in equilibrium (mg L^−1^)	
		Q_m_: The maximum presence of the capacity adsorption (mg g^−1^)	
		K_L_: Constant equilibrium study (L g^−1^)	
Freundlich	${\text{Log}}\left( {q_{e} } \right){\text{ = log}}\left( {K_{F} } \right){ + }\frac{1}{n}{\text{log}}\left( {C_{e} } \right){ (12)}$	Q_e_: Capacity adsorbed study (mg g^−1^)	^16^
		C_e_: Equilibrium concentration study (mg L^−1^)	
		K_F_: The constant present of adsorption capacity ((mg g^−1^)(L mg^−1^))	
Temkin	$q_{t} = B_{1} \ln \left( {K_{T} } \right) + B_{1} \ln \left( {C_{e} } \right){ (13)}$	b and K_T_: The constants Temkin model study respectively (J mol^−1^) and (L g^−1^)R: Constant of the Gas present (J mol^−1^ K^−1^)	^16–19^

### Advanced statistical physics

The simulation of the isotherm experimental data was performed using the software ORIGIN (version 2018) in all figures present. The choice of the most relevant model(s) for understanding the Pb (II) adsorption process onto the Cs material depended on the values and correlation coefficient R^2^ are provided in the following tables.

### Computational methodology

Calculations for density functional theory (DFT) using the jugar and Maestro Schrodinger to determine the optimal structures of the resin polymeric. The computations were performed using the 6-311G++(d,p) higher-order basis set and the B3LYP technique. The optimal structure carried out the geometrical parameters as well as other molecular characteristics like HOMO–LUMO, MEP, thermodynamic patterns and interction energy.

## Results and discussion

### Chemical characterization party

#### EDX and SEM analysis of adsorbent

**Figure 1:**
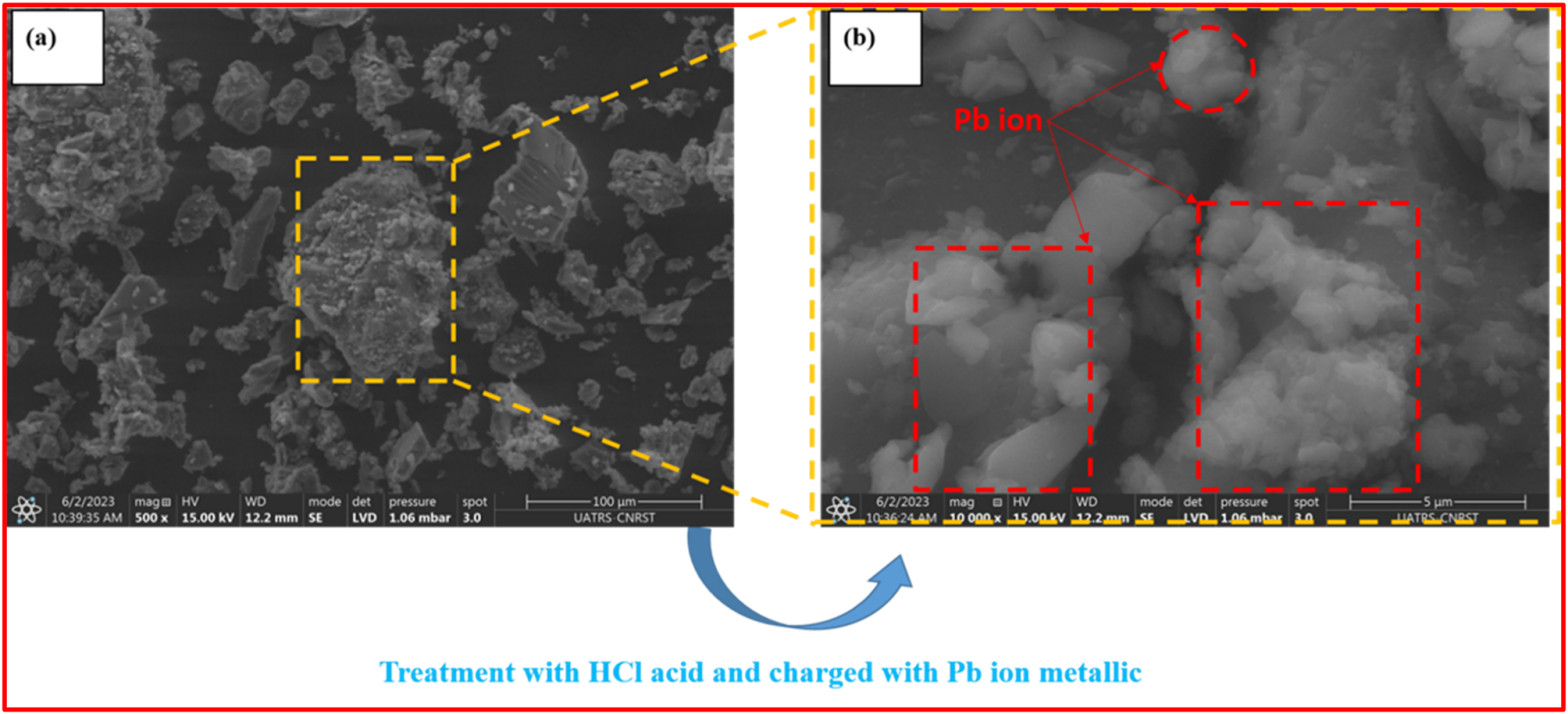
SEM of the resin without washing with acid HCl (**a**) after loaded with Pb (II) (**b**).

Each SEM obtained of the adsorbent resin polymeric, reveals the presence of strong white surfaces and before adsorption, which can be attributed to the adsorption process facilitated by the addition of HCl, the exploited results have already been published in a previous work^15^, after acid washing of the resin (Fig. 1a), and after Pb (II) treatment of the resin is presented in Fig. 1b. The different images of the adsorbent resin polymeric (Fig. 1a) polymeric reveal the presence of porosity on the surface morphology of the resin polymeric, indicating the presence of microcavities and irregular molecules, which is advantageous for this technique. However, due to lead ion (II) adsorption, strong white clouds arise in Fig. 1b.

**Figure 2:**
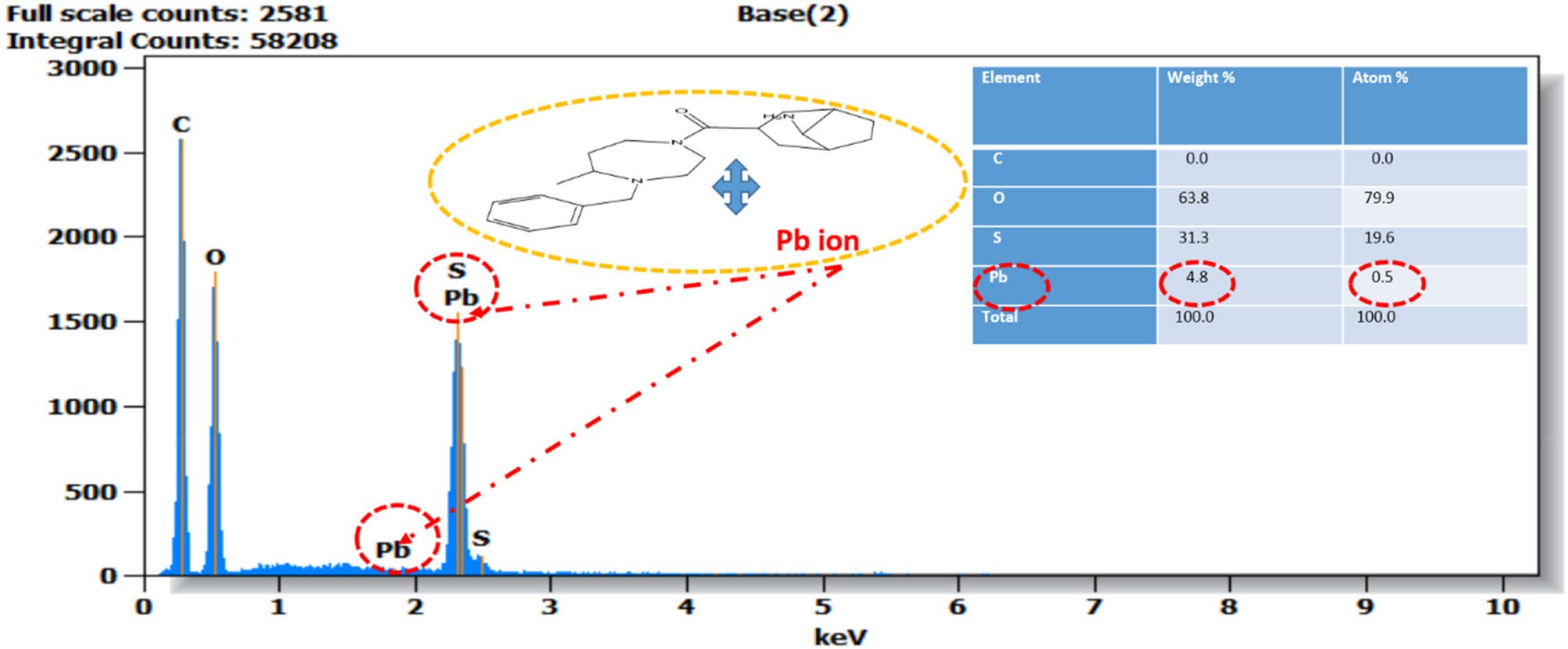
The image presents the EDX of the organic adsorbent polymeric resin after washing it with HCl and lead metal loaded Pb^2+^ (Resin + Pb^2+^).

The diagram diffraction EDX obtained in Fig. 2 which proposed this technical process of the metal Pb (II) on cationic resin organic polymeric material can provide a porous and irregular structure, which is favorable for the efficient diffusion of metal ions, particularly lead (Pb) ions.

The main purpose is to investigate the various elemental compounds present in the polymeric adsorbent the resin polymeric after treating it chemically with the metal ion Pb^2+^ are present in Table 4.

**Table 4:** Percentage mass of different chemical compositions available on the morphology of the adsorbent resin polymeric.

Elementary	C	O	Na	Si	Pb
Resin polymeric	8.0	1.6	0.2	0.3	4.2
Resin + HCl	5.8	–	0.2	0.2	10
Resin + Pb^2+^	5.8	0.5	0.2	0.2	10

The crude (resin polymeric) brut and (resin polymeric with HCl) polymeric have higher percentage carbon compared to the untreated polymeric. The evanescence of different elements as well as (Pb (II) and O) observed after treatment of this adsorbent polymeric.

#### The analysis of the thermostability of the adsorbent (GTA/GTD)

**Figure 3:**
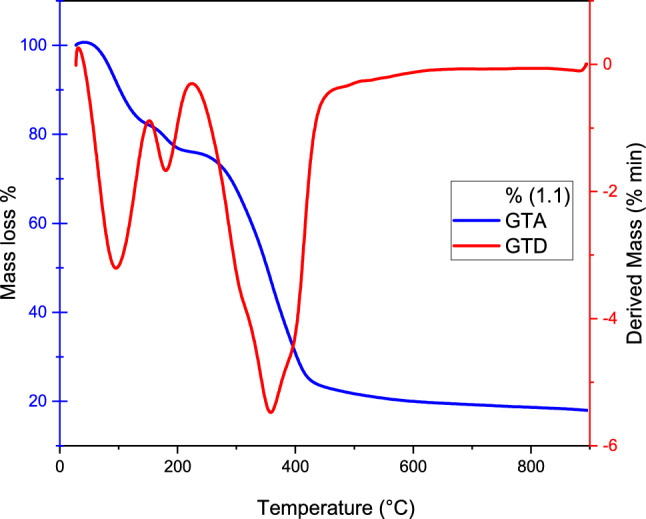
GTA and GTD thermogram of the commercial adsorbent resin polymeric and reed adsorbed filter.

Gravimetric analysis was used to conduct the thermal stability analysis of the cationic organic adsorbent polymeric resin and the filter adsorbed with Pb(II) metal ion. The study involved subjecting the samples to a temperature ramp of 10 °C min^−1^. The study involved exposing the samples to a temperature ramp of 10 °C min^−1^, which resulted in the obtaining of mass change percentages and their derivatives with respect to temperature, The range between 293 and 1073 K was determined, and the results are shown in Fig. 3.

The cationic adsorbent polymeric resin experienced a mass loss at approximately 373 K, which can be attributed to the evaporation of water. The resin has an estimated water content of around 5.7, which may fluctuate based on the ambient air's relative humidity. In the temperature range of 250–450 °C, there is a noticeable event that indicates thermal degradation of the cationic adsorbent polymeric resin. It is noteworthy that the peak at 318 °C and narrow shape indicate the degradation of the pure resin material. The initial mass accounts for approximately 0.84 of the residues obtained at temperatures above 800 °C. The thermal stability of pure materials, including the cationic adsorbent polymeric resin, can be influenced by factors such as surface morphology or sampling, which is noteworthy. The extraction from reed was used for similar analyses, with the water content estimated to be 5.5. The maximum derivative peak was observed at 300.33 °C. Residues obtained at temperatures exceeding 850 °C approximately represent 1.8% of the mass initial.

### Impact of contact time

The kinetic study of the removal metal Pb (II) by the cationic beads of the adsorbent organic resin polymeric was carried out. The highest results are presented in Fig. 3.

**Figure 4:**
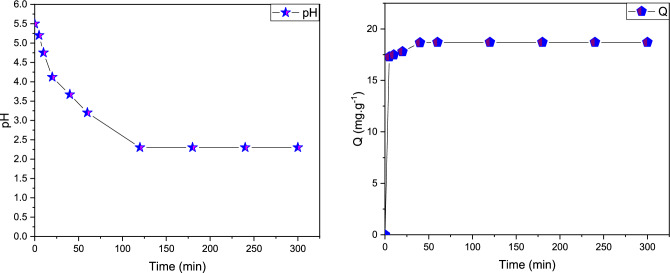
The evaluation present in the concentration of Pb (II) metal as a function of contact time and pH for the cationic resin polymeric: [Pb (II)] = 20 mg L^−1^, T = 298 K, V = 100 mL and m = 0 0.1g.

It was observed that within the initial five minutes of contact with Pb(II) metal ions, the adsorption process started, and it reached equilibrium after approximately 40 min. These results are consistent with findings reported in the literature for the adsorption of Pb (II) ions on other types of organic resin adsorbents^20–22^. The simultaneous evolution of the pH values of the metal solution and adsorption capacity (Qe) study as a function of time can be seen in Fig. 4.

**Figure 5:**
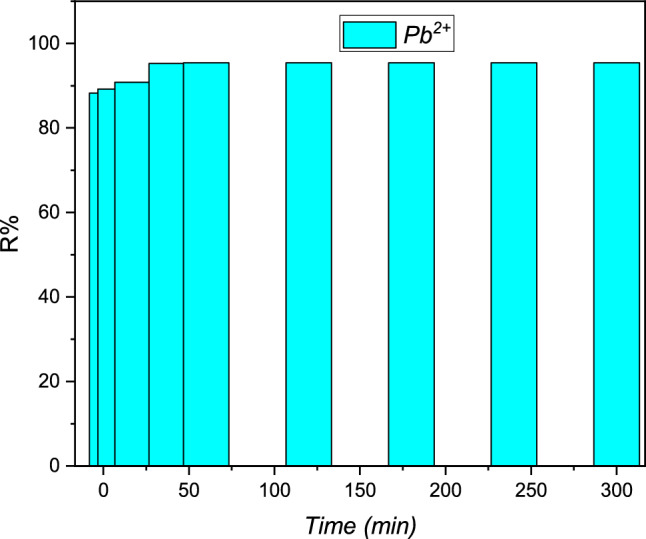
The evaluation study in the yield adsorption study of the Pb (II) as a function of the contact time for the resin polymeric: C = 20 mg L^−1^, T = 298 K, V = 100 mL, m = 0.1 g and pH = 5.5.

In fact, the results presented in Fig. 5 show that higher adsorptions yield of Pb (II) are around 95.41% during the first 20 min. This indicates a high affinity of the adsorbent organic resin polymeric for the lead cations simultaneously. Furthermore, the extraction yield increases with time and reaches practically equilibrium after 40 min with a value of 99.53% for the metal Pb (II) ions.

The adsorption capacity was better at around 40 min of Pb(II), as evidenced by the curves in Fig. 5 There is also a first phase, which is quickly followed by a little amount of posterior adsorption. The metal ions of lead Pb (II) gradually occupied the sites of adsorption by the organic resin polymeric, according to this kinetic research and the curves in Fig. 5, with equilibrium landing was established, which corresponded to the resin bead platform, with a saturation capacity of Q _max_ (Pb (II)) = 18.70 mg g^−1^. The rise in metal ions in the Pb (II) adsorption was accompanied by a drop in pH from 5.5 to 2.32. The results are very similar to those obtained in the first investigations using the metal of Pb^2+^ on hausmannite magnetic nanoparticles^23–26^.

### Effect of the mass

The study was performed with increasing masses ranging from 0.05 to 2 g. Each mass was placed in a metal solution of Pb (II) of 20 ppm at pH = 5.5 at 298 K.

**Figure 6:**
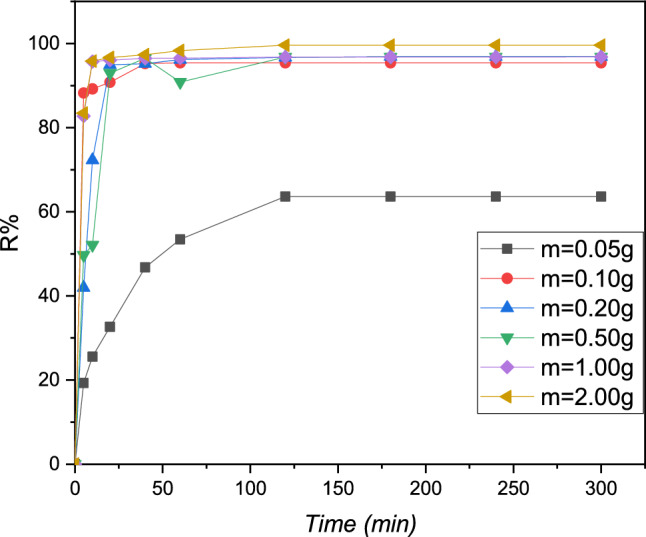
The cationic resin was used to determine the concentration of Pb (II) with a function of time at various masses study, pH = 5.5, [Pb (II)] = 20 mg L^−1^, T = 298 K, and V = 100 mL.

The results demonstrate that as the adsorbent mass grows, the adsorption contact decreases, however, the adsorption effectiveness of the inorganic pollutant Pb (II) increases in tandem with the increase in resin mass from 0.05 to 2.0 g. For the mass of 1.0 g of the cationic resin utilized is present in Fig. 6, the curves showed that the proportion of inorganic pollutants removed from Pb (II) is entirely qualitative (100 percent). These findings show that increasing the resin mass leads to an increase in the number of different sites studied for the coordination of the inorganic pollutant Pb (II), which enhances the process of metallic de-pollution of the solution investigated^27^. This number was favored as the optimum mass for economic dimensions, with a mass of 0.1 g of the adsorbent organic resin polymeric supplied at a 98 percent adsorption rate.

### Effect of pH

**Figure 7:**
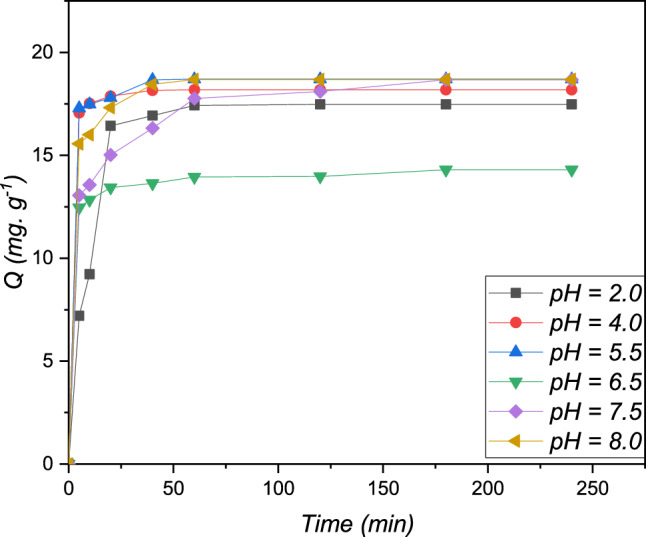
The evaluation study in the binding capacity of Pb (II) metal as a function of the contact time at different pH for the cationic resin: C_0_ = 20 mg L^−1^, T = 298 K, V = 100 mL, m = 0.1 g.

In the adsorption process, the potential hydrogen value of the metallic Pb (II) is a critical factor. Figure 7 shows the evaluation of the Pb (II) study adsorption count on the resin polymeric with a function of time for various potential hydrogen values.

**Figure 8:**
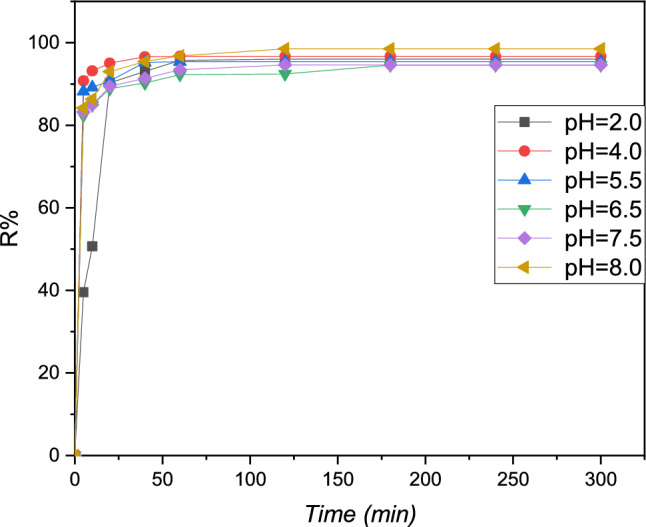
Evaluation of the percentage of removal of Pb (II) as a function of time at different pH, m _resin_ = 0.1 g, V = 100 mL, T = 298 K, and [Pb^2+^] = 20 mg L^−1^.

The results obtained show that obviously the average adsorption of Pb (II) was average in acidic conditions with the potential hydrogen of 2 and 4, where the adsorption count did not transcend 65%. The result obtained can be explained by the attendance of the amine found protonated in the resin, which decreases the interaction between Pb (II) and the activity of various sites on the surface molecular of the resin^28^. Figure 8 shows that the yield of Pb(II) adsorption by the cationic resin polymer is increases rapidly in the pH range (pH = 8), then stabilizes and becomes almost constant, at a maximum equal to (R percent = 84.78), at a pH between 2.0 and 8.0. These findings can be attributed either to the behavior of the adsorbent with respect to the change of pH in the aqueous phase or to the behavior of the Pb(II) cations (appearance and/or disappearance of Pb(II) species in the aqueous phase) at different valus of the pH study^29^.

### Impact of initial concentration

**Figure 9:**
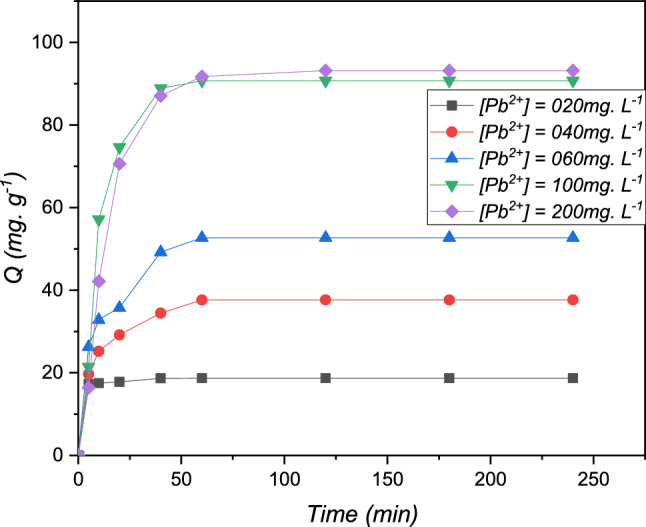
The evaluation present in the binding capacity of Pb (II) metallic as a function of time, presents at different concentrations for the resin polymeric: T = 298 K, V = 100 mL, m = 0.1 g and pH = 5.5.

The technical adsorption study of metallic pollutant of Pb (II) on the resin polymeric was studied at different concentrations (20, 40, 60, 100 and 200 mg L^−1^). The results are summarized in Fig. 9. The adsorbed rising of the metal Pb (II) at different concentrations study is rapid reaches saturation at about twenty minutes. A rapid increase in the maximum capacity study of the resin polymeric to the research metal Pb (II) present from the ionic solution was observed at the beginning for rising concentrations present, corresponding to the crossing to the acquired curve. Analysis overhead of high concentration present (100 mg L^−1^) of the metal lead (II) study, a saturation corresponding a plateau to the resin polymeric saturation is 93.18 mg g^−1^ present in Fig. 9. This is due to the depletion of numerous active sites on the molecular level of the resin^30^.

The adsorbent resin polymeric is loaded with metallic saturation and reaches the capacity binding of 93. 18 mg g^−1^ for Pb (II).

This can be explained by the fact that at low concentrations, the ratio of the active sites of the cationic resin to the total metal pollutant ions in the solution is high, and therefore all inorganic ions may be retained by the adsorbent and eliminated from the solution. However, at high concentrations, the driving force, due to the stronger concentration gradient, and quantities of Pb (II) adsorbed per unit mass of the resin, q_e_, are greater, which causes saturation of the resin. Cationic and, as a result, a quantity of ions remains free in the solution, leading to a low yield^28^.

As a result, it can be concluded that the adsorbent of the cationic resin is more effective for wastewater with low concentrations of metal ions.

### Impact of temperature

**Figure 10:**
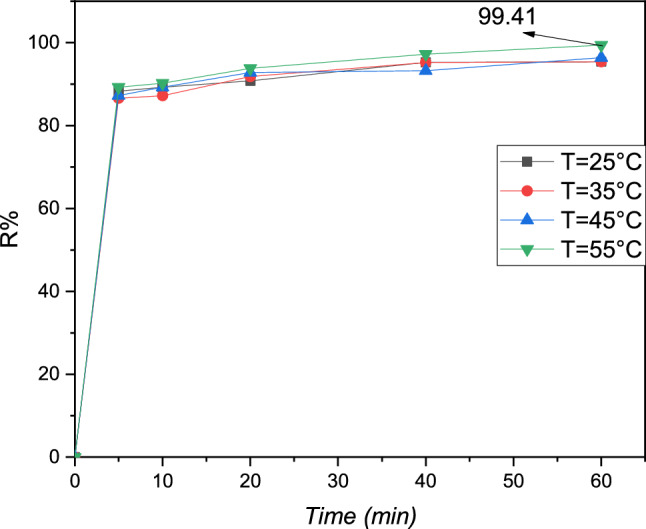
Evaluation in technical process of Pb (II) as function of T(K) of cationic adsorbent resin polymeric: C = 20 mg L^−1^, pH = 5.5, V = 100 mL, m = 0.1 g and t = 60 min.

The impact of the temperature study on high-capacity contact of this Pb solution on the adsorbent resin polymeric investigated utilized 0.1 g of the Pb (II) ions metallic present in a solution of the phial 100 mL exploited, with the door adsorption out at the gauge temperatures from 298 to 328 K in Fig. 10. the capacity contact of this solution metallic on the slightly polymeric augment with the augmentation temperature, indicating that the phenomenon process of the metal Pb (II) on the adsorbent polymeric could be an the endothermic phenomenally^29^.

Figure 10 shows the variation in efficiency as a function of temperature.

According to Fig. 10, the percentage of lead elimination is augmented slightly from 95.91–99.41% for Pb (II) when the T(K) varies in the midst 298–328 K can be obtained.

These obtained results are indicated that the adsorption endothermic process can be belayed by this fact study of the summit temperature in partiality of the agglomeration present of the metal Pb (II) on this adsorbent morphology of the cationic polymeric.

### Kinetic party of adsorption study

The adsorption phenomenon is determined using two pseudo 1st/2sec, Bangham, and Elovich models to establish the contact molecular of the adsorption process due to the Pb (II) ion metallic on adsorbent organic resin.

#### 1st order model

**Figure 11:**
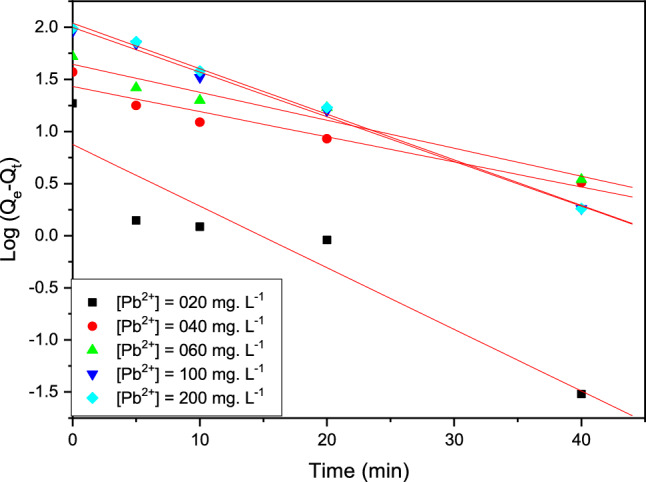
The technical adsorption rate of Pb (II) on the resin polymeric beads was evaluated using a pseudo-first-order model.

As shown in Eq. (4), the adsorption of this technical kinetic model is clear in linear form present in Fig. 11, (Table 6).

#### 2sec order model

**Figure 12:**
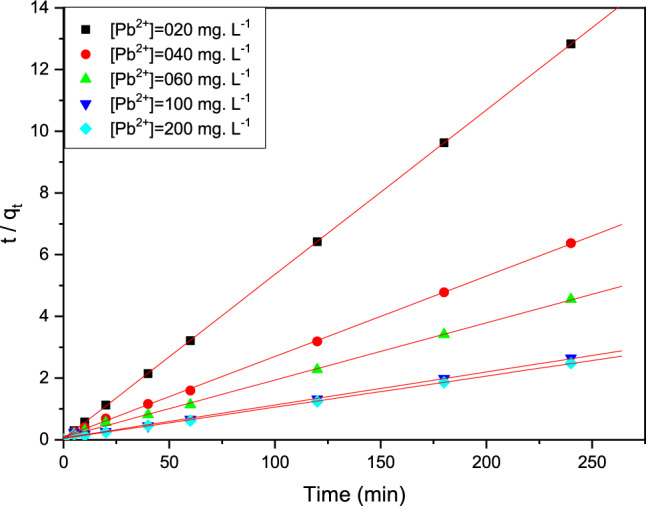
Pseudo-second order kinetic model of Pb (II) adsorption on the cationic resin beads.

As shown in Eq. (5), the adsorption of this technical kinetic model is clear in linear form present in Fig. 12, (Table 5).

**Table 5:** Parameters present of the two kinetic pseudo study as 1st/2sec models of Pb (II).

Kinetic	[Pb^2+^] mg L^−1^	q_e-exp_ (mg g^−1^)	q_e-calcul_ (m g^−1^)	k (min^−-1^)	R^2^
Pseudo 1st order	020	18.7	07.41	0.138	0.940
	040	37.67	26.91	0.055	0.970
	060	52.71	43.65	0.060	0.976
	100	90.72	102.33	0.092	0.997
	200	96.75	112.20	0.092	0.997
Pseudo 2sec order	020	18.7	18.87	0.1300	1
	040	37.67	38.46	0.0072	0.9997
	060	52.71	55.55	0.0040	0.999
	100	90.72	99.00	0.0017	0.998
	200	96.75	99.00	0.0023	1

#### Intraparticle diffusion model

**Figure 13:**
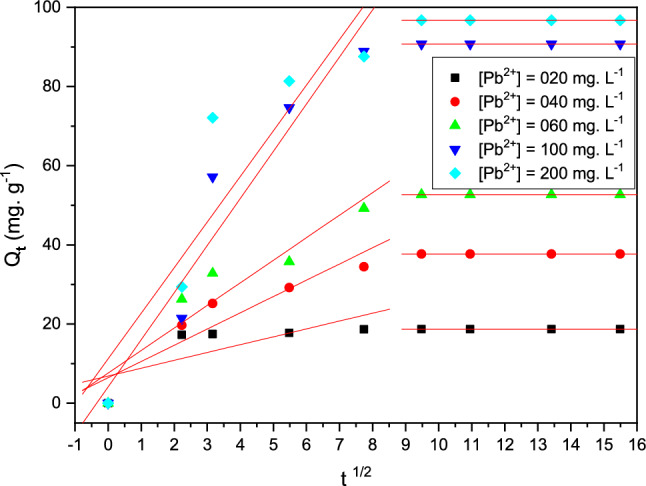
Modeling of the kinetics adsorption of Pb (II) metal ions by the model using intraparticle diffusion present.

The intraparticle diffusion model, also known as the Weber and Morris model^31^, has been investigated to demonstrate the Pb (II) diffusion process that happens during the adsorption phenomenon between Pb (II) metal and cationic resin^32,33^. The equation is shown in the following tables. This model's modeling of experimental results can exhibit multi-linearity, indicating the presence of multiple stages in the adsorption process. The 1st stage entails reducing the cationic resin adsorption via external surface diffusion. The 2sec corresponds to the slow adsorption of the resin polymeric, the phenomenon is limited by intraparticle diffusion. The third stage corresponds to a saturation state in which the absorption capacity does not change any more. In addition, if only intraparticle diffusion is involved, the function q_t_ = f (t^1/2^) crosses through the origin^34^. The higher the intercept, the more this phenomenally process is controlled by the external diffusion phenomenon. The experimental analysis data were also studied using the higher model intraparticle diffusion present. Figure 13 and Table 6. Show the results achieved.

**Table 6:** Parameters study of the intraparticle diffusion.

Adsorption of A®IRC-50	[Pb(II)] (mg L^−1^)	Time (min)	K_i_	X_i_	R^2^
Pb(II)	20	< 60	1.98	06.86	0.55
		> 60	0.57	18.7	0.83
	40	< 60	4.10	06.43	0.88
		> 60	0.65	37.67	0.82
	060	< 60	05.70	07.62	0.87
		> 60	0.63	52.71	0.76
	100	< 60	11.90	04.10	0.99
		> 60	0.38	90.72	0.63
	200	< 60	11.52	11.19	0.92
		> 60	0.76	96.75	0.73

Note that all the correlation coefficients of R > 94% for the intraparticle diffusion model, the trend curves obtained do not manage to pass through the origin of the marker. This may be due to the difference of mass transmit in the first and last adsorption step, corresponding some degree of the layer boundary control, which implies that diffusion intra-particle is not just the step speed control^34^.

According to the results obtained in Fig. 13, it is possible to split into two successive stages in the phenomenon of adsorption of lead on the cationic resin: A first stage of the adsorption is limited by the diffusion of the outer surface, which explains the results represented during the adsorption where the formation of a crown was observed. The second step corresponds to an equilibrium state in which the absorption capacity does not change (formation of a plateau). In the case of adsorption of lead on cationic resin, the diffusion constant k_i_ is higher.

The mathematical modeling of the experimental results obtained according to the intraparticle model can group together a multi-linearity which shows the existence of the two successive stages of the phenomenon of adsorption. The first stage consists of a limitation of adsorption by the diffusion external, while the second stage involves a state of the saturation equilibrium where there is no further change in adsorption capacity. Indeed, the straight lines obtained do not pass through the origin of the reference as shown in Fig. 13. Moreover, if only intraparticle diffusion is associated with the phenomenal process, the line qt = f (t^1/2^) passes the origin^34^.

#### Elovich's model

**Figure 14:**
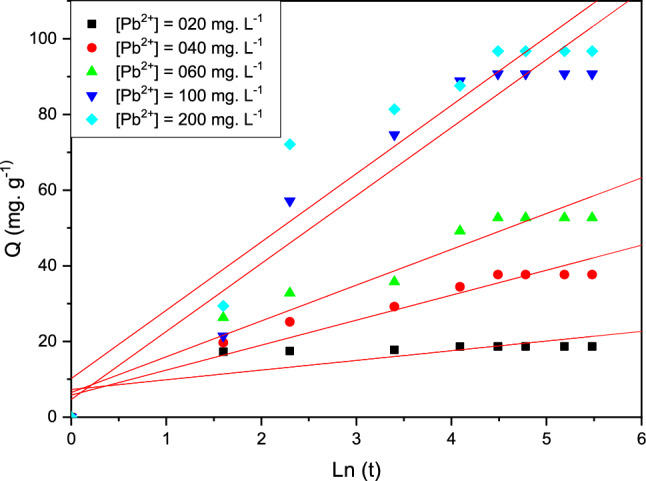
Elovich kinetic model for the adsorption of Pb (II) on the cationic resin beads.

This present Elovich’s equation was exploited to characterize this adsorption technic of the chemisorption rate which descends exactly with the increase in the capacity of the metallic solution is present in Fig. 14, Table 7.

**Table 7:** Kinetic parameters of the Elovich present.

Parameters	[Pb(II)] mg L^−1^	Α	Β	R^2^
[Pb (II)]	020	6.75	0.39	0.769
	040	0.36	0.15	0.961
	060	0.19	0.10	0.967
	100	0.06	0.05	0.961
	200	0.08	0.05	0.949

The coefficient of determination values of R^2^ are close to unity for all the concentrations studied are present in Table 7, which confirms that the adsorption of micropollutants Pb^2+^ by the resin polymeric is likely to be dominated by a process of the type of chemical reaction d adsorption (chemisorption)^34^.

#### Bangham model

**Figure 15:**
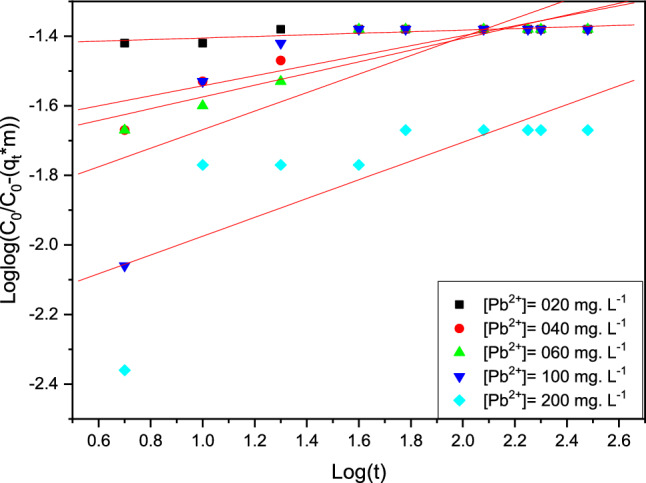
Modeling of the adsorption kinetics of Pb (II) ions by the Bangham model.

Bangham’s kinetic model investigates the adsorption process's slow pore stage diffusion, Fig. 15 and Table 8.

**Table 8:** The kinetic parameters of the Bangham study.

Pb concentration	ɑ	K_B_	R^2^
[Pb] = 020 mg L^−1^	0.02	55.66	0.80
[Pb] = 040 mg L^−1^	0.14	42.92	0.87
[Pb] = 060 mg L^−1^	0.17	40.42	0.91
[Pb] = 100 mg L^−1^	0. 26	33.43	0.73
[Pb] = 200 mg L^−1^	0. 27	24.52	0.75

For the organic polymeric resin with metal ions (Fig. 15), the linearity of the curve obtained and the different values of the correlation coefficient were unstable and less than 0.9, whereas resins not matched with the Bangham model to inorganic micropollutants by Pb (II) ions had values of the correlation coefficient that were stable and less than 0.9 in Table 8. These data clearly indicate that the adsorption of the micropollutants of the Pb (II) ions on the resin is not controlled by diffusion into the pores. The diameter and number of these are not closely related to the type of polymerization.

### Isotherm party of adsorption study

The adsorption isotherm is of great value in how the metal ions are distributed in the midst the solution metallic and the resin polymeric of at saturation state, which is fundamental for optimizing compensate of the resin. Langmuir, Freundlich, and Temkin^15,35–37^ took into account three different isotherms in the current investigation.

#### Langmuir phenomenally

The Langmuir isothermal model is instituted on the premise that adsorption occurs at a single homogenous location on the resin ground^38,39^. The Langmuir model was used to calculate the results of the various adsorptions for Pb (II) metal ions on the resin polymeric.

#### Freundlich adsorption isotherm

**Figure 16:**
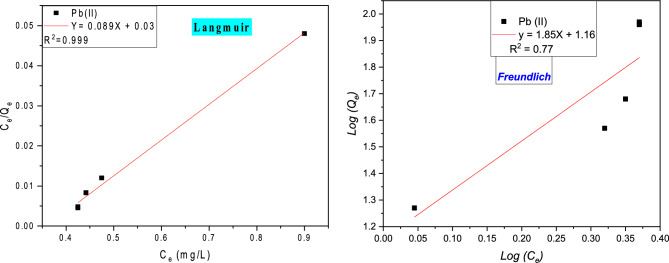
Isothermal of two models (Langmuir/Freundlich) for this phenomenal process of lead Pb (II) on the resin organic beads.

Freundlich hypothesized that the resin polymeric captures are heterogeneous and multilayers^40,41^. Table 9 shows how to express this isotherm equation in linear form can be present in Fig. 16.

**Table 9:** Parameters of the model’s study.

Langmuir	Q_mexp_(mg g^−1^) 93.18	Q_mcal_ (mg g^−1^) 11.23	∆R (%) 7.26	K_L_ (L mg^−1^) 2.97	∆G^0^ (kJ mol^−1^) − 21.40	R^2^ 0.999 ≈1
Temkin	B1 61.82	∆Q (kJ mol^-1^) 24.95		K_T_(L mg^−1^) 1.19		R^2^ 0.945
Freundlich	1/n 1.85	n 0.54		K_F_(L mg^−1^) 14.45		R^2^ 0.77

Table 9 below is show that the isotherm Langmuir fits the experimental value precisely (R^2^ = 0.999). This isotherm was found to have a single-layer adsorption capability of 42.37 mg g^−1^ for Pb(II), with a little relative variance of 7.83 percent Pb(II), this researched value is drawn close to the worth of the maximum experimental analysis adsorption phenomenally amount experimental worth's.

#### Temkin study isothermal

**Figure 17:**
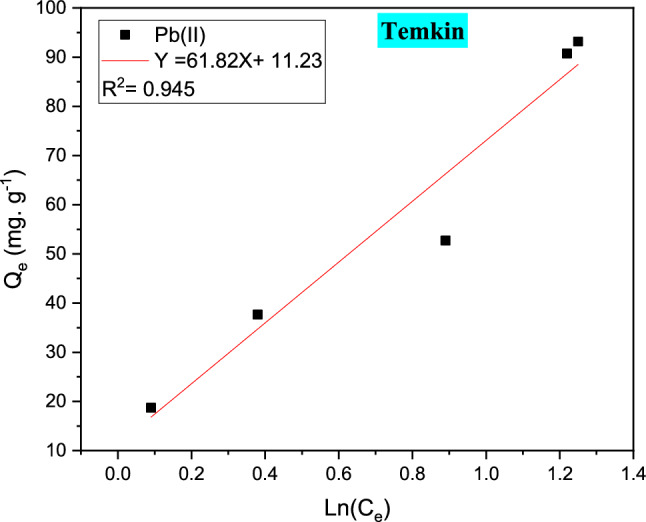
The Temkin study isothermal of Pb (II) ions adsorption study on the organic resin polymeric.

According to this isothermal, the heat of metal ion adsorption in a metallic solution of Pb (II) linearly refused with encasement due to metal-resin interactions is presents in Fig. 17, the phenomenal technique of adsorption is realized by an allocation of ordered energies binding to energy higher^42^. The equation in Table 9 can be used to give the linear form of this isotherm study.

The concentration of the cationic resin at saturation (mg L^−1^), q_e_ is the adsorbent capacity adsorbed at saturation (mg g^−1^), RT. ΔQ = B_1_ where T is the temperature (K) also R is the typical gas constant which is defined by (8.314 J mol^−1^ K^−1^) and again ΔQ is the diversity of the adsorption energy of the aqueous solution.

Various parameters can be determined using a linear present plot of the Q_e_ vs C_e_, as shown in Fig. 17. The heat of adsorption is related to the constant ΔQ. Table 9 shows the different values of A and B_1_. As a result, the physisorption process of Pb (II) on the cationic resin can be summarized by the value of (Q), which is larger than 40 kJ mol^−1^^43^.

### Thermodynamic party studies

They exploited a diversity of the factors thermodynamic, such as change present ΔG°/ΔH°/ΔS°, In this part, we have used in the same way that is used by researchers^26^.

**Figure 18:**
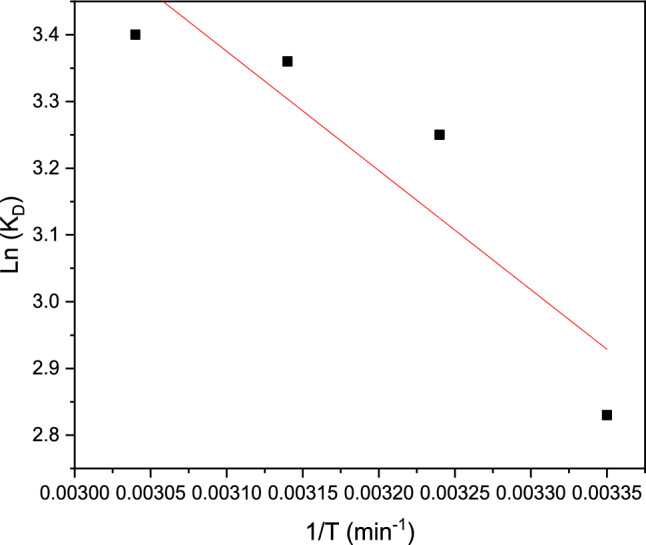
The Van 't Hoff present of the Pb(II) adsorption curve on the adsorbent resin polymeric.

The different parameters present in a two-energy study, such as ΔH° and ΔS°, determine the rates and various relationships are present in Fig. 18. As can be shown below in Table 10.

**Table 10:** Thermodynamic parameters.

T (K)	1/T (K^-1^)	Ln(K_L_)	ΔG° (kJ mol^−1^)	ΔH° (kJ mol^−1^)	ΔS° (J mol^−1^ K^−1^)
298	0.00335	2.83	− 9.78	14.85	17.00
308	0.00324	3.25	− 9.58		
318	0.00314	3.16	− 9.44		
328	0.00304	3.34	− 9.27		

The different values of ΔH ° present are major than 0, which show in this study part that this Pb(II) metallic technic phenomenally on the adsorbent polymeric is phenomenally endothermic^44^. The positive values of ΔS indicate an increase in disorder at the resin/metal interface of this cationic polymeric system, which is studied in terms of energy excited^45^.

#### Mechanisms possible present of adsorption

**Figure 19:**
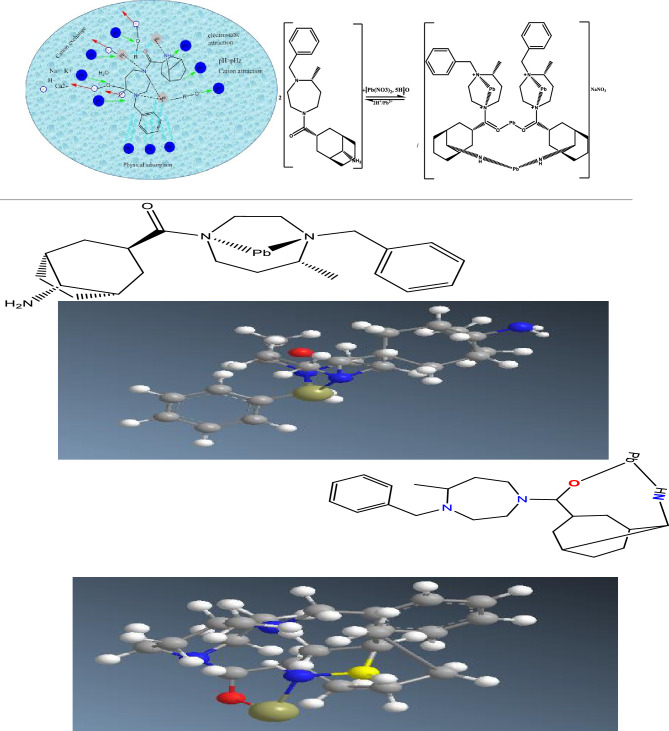
Ionic mechanisms and electrostatic interaction.

The cationic characteristics of the metal Pb (II) have been observed to result in charge delocalization. These cations can be found in ketones or nitrogen environments^46^. However, they are more frequently observed in nitrogen atoms. At pH values higher than the point of zero charge (pH_ZC_) values of the adsorbent, the ketone and amine groups are available for the adsorption of positively charged Pb (II) ions. In this pH range, the adsorbent's morphology carries a negative charge due to the presence of Pb (II) and NH_2_ groups. The optimum pH range for adsorption of Pb (II) is determined by various factors and experimental conditions. As shown in Fig. 19.

### Computational

#### Maps of FMO and electrostatic potential analysis

**Figure 20:**
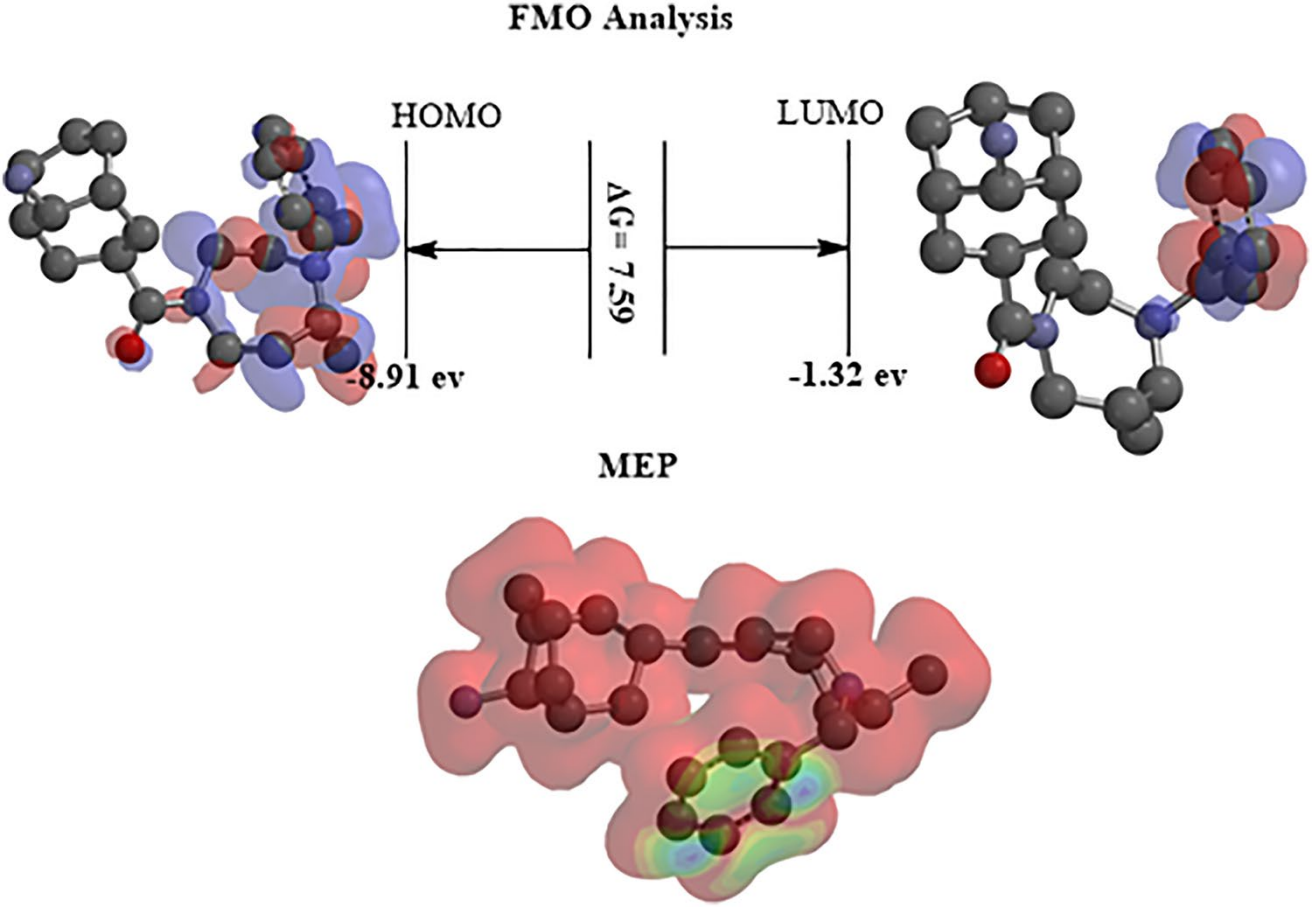
FMO and MEP maps for the resin polymeric at T-DFT/6.311G.

Conceptual density functional theories (DFT) can be used to support the understanding of the adsorption mechanism for the resin polymeric. T-DFT. B3YlP/6311G quantum chemical calculation was used to analyze FMO. The highest occupied molecular orbital (HOMO) and lowest unoccupied molecular orbital (LUMO) play a decisive part in determining the chemical behavior of a molecule the resin polymeric. HOMO and LUMO are shifted between bicyclononanamine and phenyl ring along the molecular skeleton (Fig. 20). The plot of HOMO and LUMO orbitals shows that the positive and negative regions are spread all over the molecule the resin polymeric resin. The detecting active site which chelates the Pb ions is explained by the charge allocation interaction between the resin polymeric and Pb metal ion, which is determined by the energy gap between LUMO and HOMO. A small energy gap indicates the easy charge transfer between the two orbitals and high charge transfer contact, explaining the efficiency for adsorption of the resin polymeric.

MEP is a marker for the distribution and polarization of the outer electrons in connection to the reactivity and capacity of the molecular environment to interact with H-atoms. It also offers comprehensive details on the locations of electrophilic and nucleophilic chemicals. As a result, by varying the color in Fig. 20, we may graphically establish the statistical polarity. They serve to distinguish between the polar (represented by a red charge) and nonpolar (represented by a blue charge) molecular zones. It was discovered that the green area had potential that was midway between the dual red and blue. As the distribution of colors on the MEP changes, red, yellow, blue, and green appear in increasing order (Fig. 20). The electron distribution supported that the investigated the resin polymeric able to adsorbent of the Pb metal ions based on size and shape.

#### Interaction models

**Figure 21:**
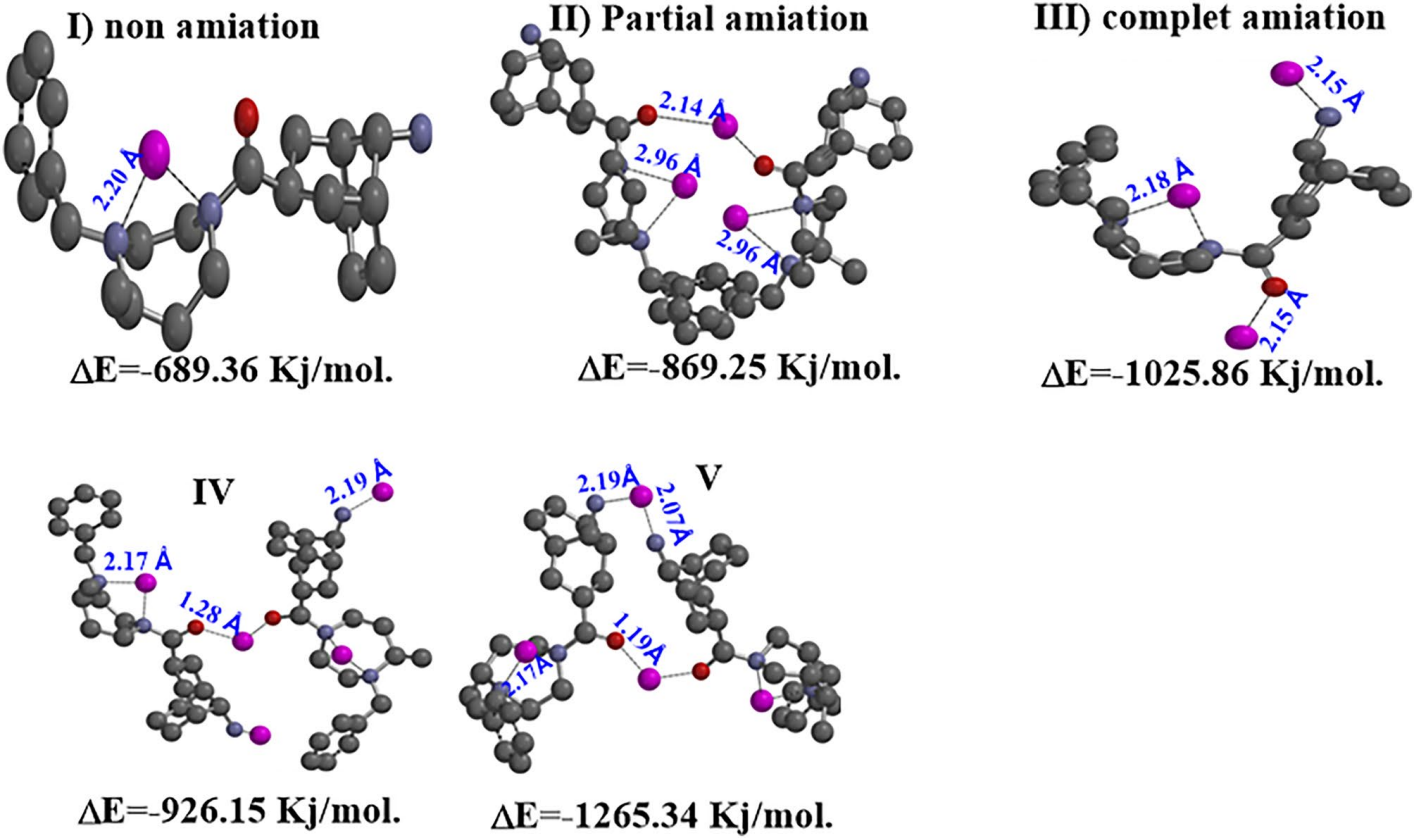
Momeric and Dimeric structures used to simulate potential basic units of the resin polymeric for coordination with Pb(II) (for clarity, hydrogen atoms are omitted from all structures).

The possible modes of coordination of the resin polymeric were further investigated using quantum chemical calculations with Pb(II) as the target metal. These calculations used monomeric and dimeric structures as models namely; for monomer non **(I)**, partial (**II**), and complete (**III**) amination and dimer partial (**V**) and complet(**IV**) amination. It should be noted that steric effects in the polymer on adsorption of the resin polymeric not considered and the focus was only on the interaction between the monomer and dimer structure of the resin polymeric and Pb. Optimized structures of possible the resin polymeric complexes **(I–IV)** are shown in Fig. 21.

For Pb(II) adsorption, the lowest ΔE values for the three monomer models I—Pb^II^, II—Pb^II^ and III—Pb^II^ is III model − 1025.86 kJ mol^−1^. The complete damnation for the dimmer model showed healthier ΔE = − 1265.35 kJ mol^−1^ than partial amination IV ΔE = − 926.15 kJ mol^−1^. Thus when thus showing a preference of Pb^II^ for N compared to O coordination, these results strongly support the experimental observation that the amination of dimer model the resin polymeric can significantly improve its adsorption capacity for the resin polymeric dimmer model. In addition, bond length in functional groups will lengthen when complete amination monomer model (**III**) is coordinated to Pb^II^ metals when compared with dimmer models IV and V.

## Conclusion

In this study, we investigated the phase solid adsorption of Pb (II) by the resin polymeric. The key findings regarding this adsorption phenomenon can be summarized as follows:

Experimental results revealed that among the metals studied, Pb (II) exhibited the highest adsorption affinity towards the resin polymeric, with time and reaches practically equilibrium after 40 min with a value of 99.53% for the metal Pb (II) ions. In other words, Pb (II) was found to be the metal that was most effectively adsorbed by the resin polymeric in the experimental conditions.

Equilibrium data were also fitted by Langmuir isotherm, so the Pb (II) ions adsorb in monolayers without any interactions between them. The maximum monolayer adsorption capacity for metal ions, using the Langmuir isotherm model equation, is equal to 11.23 mg g^−1^, with the experimental value precisely (R^2^ = 0.999). The negative values of ΔG reveal the spontaneous nature during the adsorption process of metal ions onto the resin polymeric. ΔH and ΔS positive values have proved the endothermic and randomness of the adsorption process, such as ΔG° (− 9.78 to − 9.27 kJ mol^−1^), ΔH° (14.85 kJ mol^−1^), and ΔS° (0.017 kJ mol^−1^) were determined (Supplementary Fig. S1).

Finally, in theory party, the complete damnation for the dimmer model showed healthier ΔE = − 1265.35 kJ mol^−1^ than partial amination IV ΔE = − 926.15kJ mol^−1^, on the other hand the lowest ΔE values for the three monomer models I—Pb^II^, II—Pb^II^ and III—Pb^II^ is III model − 1025.86 kJ mol^−1^.

### Supplementary Information

Supplementary Figure S1.

## Data Availability

All data generated or analysed during this study are included in this published article.
