# Supplementary figures and images for: Optimization of an experimental study of cationic Pb metal adsorption by resin polymer
Source: Sci Rep. 2023 Nov 16;13:20060. doi: 10.1038/s41598-023-46967-3 (PMC10654399; doi:10.1038/s41598-023-46967-3)

**Supplementary Material**



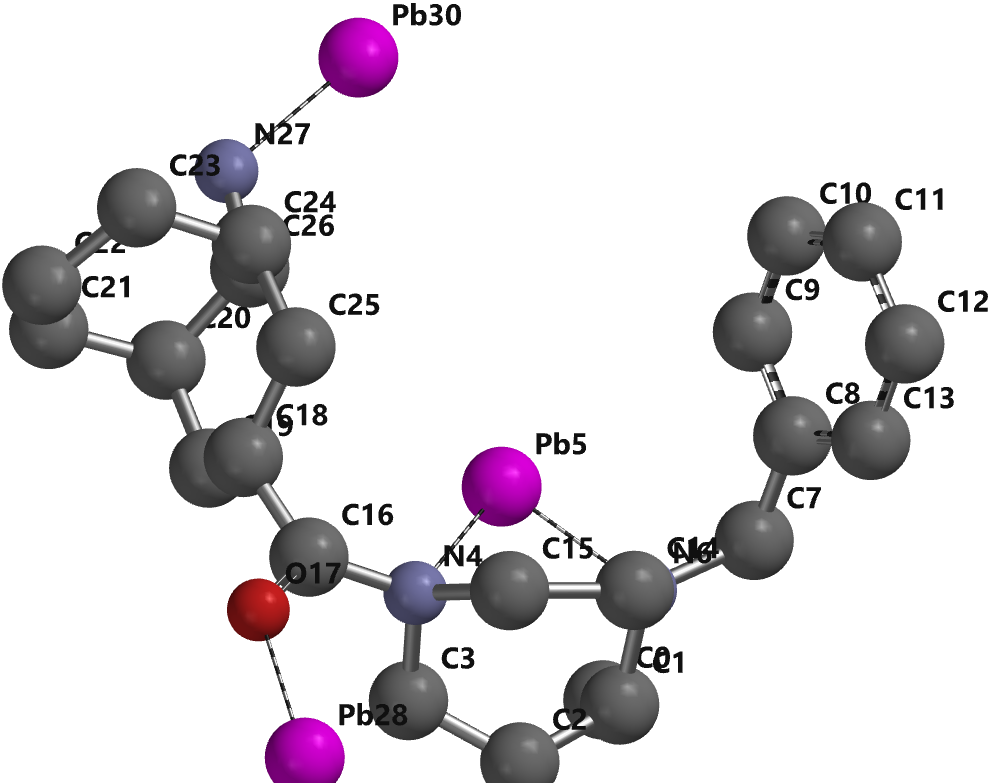


Figure S.1: numbering number for optimization of III

Supplement: Supplementary file 1 — Supplementary Figure S1. [file 41598_2023_46967_MOESM1_ESM.docx]
